# Hemophagocytic Lymphohistiocytosis (HLH) Induced by Epstein-Barr Virus in a Patient With Human Immunodeficiency Virus (HIV) Infection

**DOI:** 10.7759/cureus.93979

**Published:** 2025-10-06

**Authors:** Harshasree Seelam, Aye M Thida, Ezoza Yunusova, Robert Levy

**Affiliations:** 1 Department of Hematology and Oncology, State University of New York Downstate Health Sciences University, Brooklyn, USA; 2 Department of Internal Medicine, State University of New York Downstate Health Sciences University, Brooklyn, USA

**Keywords:** ebv hlh, hemophagocytic lymphohistiocytosis (hlh), hiv, hlh-94, hlh in adults

## Abstract

Hemophagocytic lymphohistiocytosis (HLH) is a rare, life-threatening syndrome characterized by excessive immune activation, and diagnosing it in patients with HIV is challenging due to overlapping clinical and laboratory findings. We present the case of a 69-year-old Afro-Caribbean female with advanced HIV, partial adherence to antiretroviral therapy, and Epstein-Barr virus (EBV) infection, who developed HLH. Despite initial management with antibiotics, antiretroviral therapy, and supportive care, her condition deteriorated with worsening thrombocytopenia, renal and liver impairment, and elevated inflammatory markers. The clinical syndrome was most consistent with HLH, with a calculated HLH probability score of >99%. EBV DNA levels and high interleukin-2 receptor levels confirmed EBV as the trigger for HLH. Treatment with dexamethasone, intravenous immunoglobulin, etoposide, and rituximab was initiated, but the patient developed severe pancytopenia and renal failure, ultimately passing away. This case underscores the need for a heightened index of suspicion for HLH in HIV patients and the complexities of its management, including the limitations of the HLH-94 regimen in immunocompromised individuals. Further research is required to determine the optimal treatment approach for HIV-associated HLH.

## Introduction

Hemophagocytic lymphohistiocytosis (HLH) is a rare and life-threatening syndrome characterized by excessive immune activation. Its pathophysiology is thought to stem from uncontrolled T-cell activation, which, in turn, drives widespread myeloid cell activation through a state of hypercytokinemia. Although the mechanisms by which T-cells bypass immune checkpoints and promote dysregulated cytokine release remain incompletely understood, this hyperinflammatory response results in significant multiorgan dysfunction [1].

HLH is broadly classified into familial and acquired forms. Familial HLH is typically associated with inherited mutations affecting cytotoxic function, whereas acquired HLH arises secondary to external triggers [2]. Among these, malignancies are the most commonly implicated, followed by infections, autoimmune or rheumatologic diseases, drug hypersensitivity reactions, and transplant-related complications [3]. Notably, among infectious causes, Epstein-Barr virus (EBV) has been most frequently associated with the onset of HLH. In immunocompromised individuals, such as those with HIV, suppressed cytotoxic immune surveillance can lead to reactivation of latent EBV, precipitating a disorganized response through excessive proliferation of EBV-infected lymphocytes, triggering HLH. Patients with HIV who develop HLH are typically severely immunocompromised and more often triggered by opportunistic infections such as disseminated histoplasmosis, cytomegalovirus (CMV), or tuberculosis, whereas HIV-negative patients more commonly develop HLH in the setting of autoimmune diseases or malignancies. Diagnosis in HIV-positive individuals is often delayed due to overlapping clinical features with AIDS-related infections, leading to higher mortality and greater treatment complexity, including management of co-infections and potential immune reconstitution inflammatory syndrome (IRIS). In contrast, HIV-negative HLH patients may have more straightforward presentations and respond better to standard HLH therapies when the underlying trigger is promptly addressed.

The clinical presentation of HLH is often nonspecific and overlaps with other systemic illnesses. Common signs and symptoms include prolonged fever, hepatosplenomegaly, lymphadenopathy, cytopenias, coagulopathy, liver dysfunction, pulmonary involvement, and neurologic impairment [3]. This diagnostic challenge is further amplified in patients with HIV, where advanced disease may mimic or mask the features of HLH, complicating prompt recognition and intervention.

Here, we present a case of a 69-year-old female with advanced HIV who developed HLH following Epstein-Barr virus (EBV) infection, emphasizing the need for a heightened index of suspicion when evaluating HLH in HIV patients.

## Case presentation

A 69-year-old Afro-Caribbean female with a 31-year history of advanced HIV and partial adherence to antiretroviral therapy (ART) presented with fever, cough, diarrhea, nausea, and vomiting. On triage, she was febrile with a temperature of 101.7°F. On examination, she was slightly lethargic and had mild abdominal tenderness. Initial lab results revealed a white blood cell count of 3.96 K/uL, an absolute lymphocyte count of 0.85 K/uL, hemoglobin of 11.3 g/dL, a platelet count of 76 K/uL, aspartate aminotransferase of 136 U/L, and alanine aminotransferase of 92 U/L. Her CD4 count was 58 cells/µL. Computed tomography of the abdomen and pelvis with contrast showed enterocolitis but no hepatosplenomegaly. A workup for opportunistic infections was sent, and she was started on vancomycin, piperacillin/tazobactam, antiretroviral therapy (ART), and trimethoprim/sulfamethoxazole. However, her clinical condition deteriorated on hospital day four, with worsening mental status, thrombocytopenia, and renal and liver impairment. There were no petechiae and no evidence of bleeding or bruising on examination. A review of the peripheral blood smear showed no immature precursor cells, schistocytes, or platelet clots. Further investigation revealed high fibrinogen (208 g/L), triglycerides (582 mmol/L), and ferritin (82,756 μg/L), leading to an HLH score of 254 points, which translates to >99% probability of HLH in the setting of known immunosuppression and high fever.

Intravenous immunoglobulin 0.4 mg/kg and dexamethasone 10 mg/m² were initiated for the treatment of HIV-associated HLH. After five days of treatment, the patient’s clinical status remained largely unchanged despite some improvement in inflammatory markers. Further diagnostics revealed an elevated IL2 receptor (12,940 pg/ml) and an Epstein-Barr virus (EBV) DNA level of 56,800 copies on PCR, confirming EBV as the likely trigger for HLH. By day 13 of hospitalization, the patient continued to have fevers and elevated inflammatory markers. As part of the HLH-94 regimen, etoposide and dexamethasone were then given. The patient also received a dose of intrathecal methotrexate with steroids. In addition, rituximab was given to specifically target the EBV trigger. After one cycle of treatment, there was a transient improvement in inflammatory markers along with a decrease in EBV DNA from 56,800 IU/mL to 48 IU/mL. However, the patient developed severe pancytopenia after cycle one, refractory to granulocyte colony-stimulating factor agents (Table 1), which limited further treatment. As well, the patient also had progressive renal failure despite renal replacement therapy, leading to the patient's passing away on day 26.

**Table 1:** Overview of treatment with associated lab parameters AST: Aspartate Aminotransferase, ALT: Alanine Transaminase, LDH: Lactate Dehydrogenase, IVIG: Intravenous Immunoglobulin, Dexa: Dexamethasone, IT MTX: Intrathecal Methotrexate, N/A: Not applicable

Day of Hospitalization	Treatment	White Cell Count	Hemoglobin	Platelet Count	ALT	AST	Total Bilirubin	Creatinine	Ferritin	LDH	Fibrinogen	D-dimer
1	N/A	3.96	11.3	76	92	136	1.1	1.06	N/A	N/A	N/A	N/A
2	N/A	3.35	12.6	42	69	116	1.1	1.17	N/A	544	N/A	N/A
3	N/A	3.00	11.1	37	52	130	0.6	1.18	N/A	N/A	N/A	N/A
4	N/A	2.96	12.5	33	218	878	1.3	2.46	N/A	N/A	N/A	N/A
5	N/A	8.43	10.8	45	176	974	0.2	6.69	82,756	3,299	208	2,455
6	IVIG, dexa	8.27	10.8	31	143	911	1.7	N/A	N/A	3,537	N/A	N/A
7	IVIG, dexa	9.01	10.6	31	67	483	2.4	7.00	97,832	2,370	181	7,627
8	IVIG, dexa	6.64	8.4	43	59	401	2.8	3.92	43,252	>1,800	162	3,260
9	IVIG, dexa	5.73	8.1	25	55	300	2.6	5.92	26,252	1,281	197	1.974
10	IVIG, dexa	5.42	8.3	23	58	295	2.6	6.43	22,151	1,199	208	1,901
11	N/A	6.98	7.2	48	48	213	2.5	6.33	9,734	997	N/A	2,801
12	N/A	8.10	7.4	37	49	300	2.7	4.05	6,439	N/A	N/A	3,662
13	Etoposide, Rituximab	12.55	8.0	41	42	295	N/A	5.76	5,534	N/A	114	N/A
14	IT MTX	12.0	9.2	45	42	213	5.3	4.35	4,926	N/A	147	12,515
15	N/A	10.66	10.6	97	N/A	149	N/A	2.69	4,270	N/A	198	21,982
16	N/A	9.67	8.2	69	N/A	N/A	N/A	4.76	4,043	N/A	268	14,514
17	Etoposide	9.28	8.2	64	48	102	N/A	6.09	N/A	1,074	359	14,355
18	N/A	3.98	7.9	44	N/A	N/A	N/A	5.14	N/A	987	341	12,497
19	Neupogen	0.22	7.5	31	62	51	3.0	4.03	3,942	N/A	102	9,386
20	Neupogen	0.05	7.4	14	57	43	2.7	4.87	4,056	837	507	7,267
21	Rituximab, Neupogen	0.01	7.3	36	26	24	0.8	1.01	N/A	349	562	5,423
22	Neupogen	0.02	7.0	18	148	135	6.9	4.89	N/A	784	647	7,770
23	Neupogen	0.01	6.5	16	119	68	6.2	4.06	13,493	590	N/A	6,343
24	Neupogen	0.04	8.7	13	92	84	5.9	4.18	24,951	N/A	N/A	7,570
25	Neupogen	0.01	6.9	4	114	277	6.1	3.53	N/A	N/A	N/A	N/A
26	N/A	0.02	7.5	21	163	422	7.1	N/A	N/A	N/A	N/A	N/A

Lab Test	Reference Range
White Cell Count	4.00-10.00 per microliter
Hemoglobin	12.0-15.5 gm/dl
Platelet Count	140-440 10^3 ^per microliter
ALT	7-55 U/L
AST	8-33 U/L
Total Bilirubin	0.2-1.2 mg/dL
Creatinine	0.59-1.04 mg/dL
Ferritin	24-306 ng/mL
LDH	125-220 U/L
Fibrinogen	200-393 g/dL
D-dimer	<500 ng/mL

## Discussion

HLH is a syndrome of excessive immune activation due to the failure of the feedback mechanism of immune responses of macrophages and lymphocytes. This is either due to a genetic abnormality of perforin-dependent cytotoxicity or, more commonly, an immunologic trigger such as infection, malignancy, or an autoimmune/rheumatologic disorder [1]. Although HLH is more widely described in the pediatric population, it is being increasingly recognized in adults, with a more complex clinical presentation and underlying pathology. Most patients become acutely ill with multi-organ involvement, and mortality rates in adult populations have been reported to range from 40% to 57% [4].

HLH-94 was the first prospective international treatment study for HLH, and it is now superseded by HLH-2004. HLH-2004 notes that five out of eight criteria are required for diagnosis in the absence of a positive family history or molecular diagnosis. These criteria consist of fever, splenomegaly, bicytopenia, hypertriglyceridemia, and/or hypofibrinogenemia, hemophagocytosis, low/absent natural killer cell activity, hyperferritinemia, and high soluble interleukin-2 receptor levels [5]. Recent guidelines suggest that determining the H-score is the preferred diagnostic approach for adult patients. The H-score estimates the likelihood of HLH based on the severity of clinical and laboratory findings, with a score of over 169 indicating a significant likelihood [6]. Of note, the presence of hemophagocytosis in bone marrow aspirate or any other tissue is not absolutely necessary for diagnosis, as the finding is neither sensitive nor specific [7]. For this reason, bone marrow biopsy was deferred in our case, as the patient had already met multiple other diagnostic criteria.

Adult-acquired HLH may be more common than previously noted and appears to be underdiagnosed. This is largely due to overlapping signs and symptoms in adult patients with multiple comorbidities. Patients with advanced HIV are commonly hospitalized with fever, cytopenias, liver dysfunction, and elevated ferritin, and these findings can mimic sepsis, malignancy, or advanced HIV infection, leading to delays in diagnosis [8]. Furthermore, the HLH probability score is not specific enough to delineate a diagnosis of HLH in these patients and should be interpreted in conjunction with the clinical scenario. A high index of suspicion is required, especially when standard treatment for sepsis fails to achieve an adequate downtrend in inflammatory markers.

Our case is focused particularly on EBV-induced HLH in the context of advanced HIV. Recent studies show that HIV-positive patients with HLH have higher levels of EBV viral load compared to HIV-negative individuals [9]. Patients with HIV, particularly those with low CD4+ T cell counts, have a compromised immune system that impairs the ability to control EBV replication. EBV reactivation in this population can lead to a hyper-inflammatory response, manifesting as HLH. The combination of HIV and EBV can have a synergistic effect on immune dysregulation, leading to cytokine storm and multi-organ failure, a hallmark of HLH.

Management of HLH involves identifying and addressing the underlying cause, along with suppression of the immune response using IVIG and steroids. Additionally, chemotherapy is used in conjunction with immunotherapy to suppress cytokine-stimulated cell activation. Historically, two major treatment approaches have been described: HLH-94 and HLH-2004. Both of these regimens are largely based on pediatric populations but have been extrapolated to treat adults as well. Due to limited cases of HLH in adults complicated by extensive comorbidities as compared to children, many variable treatment approaches were reported in the literature [10]. There is consensus that early initiation of ART results in favorable outcomes in patients with HIV, but beyond that, the efficacy of other treatment modalities remains varied. As per the HLH-94 protocol, induction therapy with etoposide is indicated for malignancy-associated HLH as well as when there is clinical deterioration, as demonstrated in our case. The use of etoposide led to a severe worsening of pancytopenia in our patient, limiting further treatment. This underlines a fundamental challenge when treating adults, as these regimens are better tolerated in pediatric populations, who usually have fewer comorbidities. Cyclosporine, although included in the HLH-2004 study, had not consistently demonstrated added benefit to the affirmed efficacy of etoposide and dexamethasone, and to avoid additional toxicity, we deferred the use of cyclosporine in our patient. Targeted treatment of the presumed underlying trigger is also a key component of management. Rituximab, a monoclonal anti-CD20 antibody, is indicated when EBV DNA levels exceed 10,000 copies/mL. It causes a decrease in EBV-infected B cells and has been associated with decreased mortality [11]. As the EBV viral load in our case was over 50,000 copies/mL, rituximab was used as targeted treatment. The role of antivirals such as acyclovir in the treatment of HLH is not well established, and current consensus generally advises against their use. This is primarily because EBV exists predominantly in a latent phase within B cells during HLH, rendering acyclovir, an agent that targets the lytic phase of herpesviruses via inhibition of viral DNA polymerase, ineffective. Neither clinical experience nor available studies have demonstrated any significant benefit of acyclovir in managing EBV-associated HLH.

Treatment response can be assessed by monitoring inflammatory marker trends as well as by quantitative PCR measurements of viral DNA level. Those with higher ferritin levels were reported to have less response to induction therapy with etoposide. Those who achieved a ferritin level of 1,188 µg/L at two weeks post-induction were noted to have a better induction response [12]. A retrospective observational study by Zhang et al. found that high EBV viral DNA levels at initial diagnosis and high levels during treatment were associated with worse prognosis. Of note, the aforementioned study was based on children, and it is uncertain if the same thresholds can be applicable to adult patients. Our patient had a high viral DNA level initially and had a significant downtrend after treatment, demonstrating a response to targeted therapy; however, her course was complicated by severe pancytopenia and multi-organ failure. This demonstrates the inadequacy of the suggested immune response suppression agents even when the underlying triggers (HIV and EBV) are targeted with therapy.

Despite aggressive therapy, the prognosis remains poor in the majority of adult patients with HLH. Current recommendations from emerging studies include more diverse immunotherapies with Janus kinase (JAK) inhibition, IL-1 blockade, IFN-gamma antibody, and the use of calcineurin inhibitors [13]. Patients who experience relapse and exhibit neurological symptoms are considered for allogeneic hematopoietic stem cell transplantation (HSCT). Though HSCT showed improved survival in adult patients with EBV-associated HLH, its necessity remains controversial. A recent study by Yao et al. found that HSCT should be recommended for patients who achieved a partial response after initial treatment, while those who achieved a complete response may simply be observed [14].

## Conclusions

Hemophagocytic lymphohistiocytosis is a complex disease entity with variable presentations. Though EBV is a common trigger for HLH in children, the overlapping manifestations of advanced HIV often make the diagnosis a challenge when these conditions present simultaneously in adults. In addition, current treatment regimens for HLH are largely based on the pediatric population and are not as well tolerated in adults, thus requiring individual tailoring. The optimal approach for adult patients with EBV-triggered HIV-associated HLH needs to be further elucidated.
